# Micronized Palmitoylethanolamide, Hempseed Oil, and Maritime Pine Bark Dry Extract (Pelvipea^®^) for Pelvic Pain: An In Vitro Study for Urothelial Inflammation Treatment

**DOI:** 10.3390/cells12040616

**Published:** 2023-02-14

**Authors:** Alessandro Tafuri, Andrea Panunzio, Rita De Mitri, Federico Benetti, Elisa Gaio, Vincenzo Pagliarulo

**Affiliations:** 1 Department of Urology, “Vito Fazzi” Hospital, 73100 Lecce, Italy; 2 ECSIN-European Center for the Sustainable Impact of Nanotechnology, EcamRicert Srl, Corso Stati Uniti 4, 35127 Padova, Italy

**Keywords:** pelvic pain, urothelial inflammation, palmitoylethanolamide, hempseed oil, maritime pine bark dry extract, Pelvipea^®^

## Abstract

Urothelial inflammation plays a key role in the pathogenesis of chronic pelvic pain due to its origin in the bladder. The aim of this study was to evaluate the efficacy of a patent-pending formulation (Pelvipea^®^) composed of micronized palmitoylethanolamide (PEA), hempseed oil, and maritime pine bark dry extract in reducing urothelial inflammation, as well as the effect of each ingredient individually, in order to define the synergistic effect of the three ingredients. An in vitro bladder urothelium model composed of the T24 cell line was exposed to a conditioned media obtained by treating macrophage-differentiated THP-1 cells with different concentrations of the functional ingredients and a mixture of them in the presence of the pro-inflammatory stimulus of *Escherichia coli*. Cells exposed only to the inflammatory stimulus in the absence of pre-treatment were considered as a positive control for inflammation. The impact of each functional ingredient and their mixture on inflammation was evaluated by the determination of transcription factor NF-kB and of pro-inflammatory cytokine expression. Statistical analysis was performed using the *t*-test, comparing the mixture and the single ingredients for every condition tested. All results were reported as fold change (mean ± standard deviation), the ratio between the values obtained from the respective treatments for inflammation control. The three functional ingredients did not induce negative effects on THP-1 cell vitality. The levels of NF-kB were reduced following treatment with hempseed oil, maritime pine bark dry extract, and the mixture at all tested concentrations, and with micronized PEA from 25 to 200 μg/mL. Treatment with the mixture resulted in the lowest expression levels of interleukins (IL)-1β, IL-6, and IL-8 compared to the single functional ingredients at a concentration of 230 μg/mL, with values of 0.08 (±0.00), 0.01 (±0.00), and 0.32 (±0.01), respectively. The mixture of micronized PEA, hempseed oil, and maritime pine bark dry extract (Pelvipea^®^) at 230 μg/mL showed the best efficacy in urothelial IL-1β, IL-6, and IL-8 reduction compared with the singular components. This formulation may represent a promising therapeutic option to relieve painful symptoms originating in the bladder. However, in vivo studies are needed to confirm these results.

## 1. Introduction

Chronic pelvic pain (CPP) is a chronic or persistent pain perceived as continuous or recurrent for at least three months in structures related to the pelvis in men or women. Causes may include local pathologies such as infections, malignancy, or primary anatomical, functional, or neurogenic disease of pelvic organs. When there is no evidence of a disease accounting for the pain, the term refers to chronic pelvic pain syndrome (CPPS), which includes primary prostate, scrotal, penile, vulvar, urethral, and bladder pain syndrome, as well as other pain syndromes related to the pelvic structures [1].

The real prevalence of CPPS is not clear, mainly due to the different diagnostic criteria, the overlapping symptoms with other diseases, and missing diagnoses. Mathias et al. reported a prevalence of CPP in the United States among women aged 18 to 50 years of around 14.7%; in more than half of cases, the etiology was unknown [2]. Marszalek et al. reported a prevalence of symptoms suggestive of CPP of 5.7% in women and 2.7% in men [3].

In particular, bladder pain syndrome due to chronic cystitis such as interstitial cystitis seems to have a high prevalence in CPPS [4]. Patients might have an inflammatory reaction in the bladder, as well as in the central nervous system after insults to the bladder that produce early bladder symptoms such as urinary frequency, nocturia, and continuous or acute bladder pain [4,5,6]. Urothelial dysfunction together with local inflammatory agents might activate the inflammatory system through several interleukins (IL-1, IL-6, and IL-8) and cytokines. Additionally, chronic inflammation, impaired bladder circulation, neurogenic hyperactivity, and systemic functional disorders have been linked to CPPS and presumably interact in causing bladder-derived pain syndromes [4,5,6].

Pelvic pain syndromes have a significant impact on patient physical and psychological status, potentially leading to depression, anxiety, insomnia, and fatigue, as well as impairing working, socializing, and quality of life [7]. The management of CPPS is based on a bio-psychosocial model, which includes active patient involvement. Pharmacological and non-pharmacological interventions, such as psychotherapy, physiotherapy, drugs, botulinum toxin injection, and phytotherapy, often need to be considered together as a part of a multimodal and personalized treatment strategy [1,8,9]. Considering phytotherapy, in recent years, hempseed oil and derivates and maritime pine bark dry extract have been suggested to have important nutrition benefits for humans and useful analgesic properties. Their capacity to inhibit pro-inflammatory interleukins and cytokines has been shown and their interaction with inflammatory components such as the nuclear factor kappa-light-chain-enhancer of activated B cells (NF-kB) also reduces oxidative stress, suggesting their possible application as a treatment of acute and chronic inflammatory conditions [10,11,12,13]. Additionally, palmitoylethanolamide (PEA) is an active anti-inflammatory agent produced by microglia and mast cells, that modulates their activation. Preclinical studies showed a clinically significant reduction in the inflammatory process and chronic pain with PEA. Recently, a few studies have also demonstrated the efficacy and safety of PEA in chronic pain reduction and in controlling urinary symptoms [14,15].

In this study, we aim to evaluate the efficacy of a patent-pending formulation (Pelvipea^®^) composed of micronized PEA, hempseed oil, and maritime pine bark dry extract, as well as the efficacy of each of these components individually, in reducing inflammation in an in vitro bladder urothelium model, in order to describe the synergistic effect of the three ingredients in the formulation, in addition to identifying the minimum concentration at which an anti-inflammatory activity is present. In this context, concentrations higher than the highest concentration tested were not investigated due to cell mortality caused by the mixture at the highest concentration tested, while lower concentrations of the mixture and the individual functional ingredients were investigated for anti-inflammatory capacity.

## 2. Materials and Methods

### 2.1. Determination of the Titer in the Three Raw Materials

Evaluation of the anti-inflammatory activity was carried out on the formulation (mixture) and on the three components individually (Supplementary Table S1). Palmitoylethanolamide content in the raw material (micronized PEA powder) was determined by high-performance liquid chromatography with photodiode-array detection (HPLC-DAD) analysis. The procyanidins content in the bark dry extract of maritime pine (Pycnogenol^®^, *Pinus pinaster* Aiton bark d.e.) was evaluated by a spectrophotometric method. The dosage of total lipids contained in hempseed oil (*Cannabis sativa* L.) was measured by gas chromatography with flame ionization detection (FID) analysis.

### 2.2. Bacterial Strain

An aliquot of *Escherichia coli* (*E. coli*, ATCC^®^ 8739TM) was placed on agar plates with tryptic soy agar (TSA) medium and allowed to grow for 24 h. A single colony was harvested with a sterile inoculating loop and inoculated in liquid tryptic soy broth (TSB) medium. After 24 h at 37 °C, the concentration of bacteria (colony-forming units—(CFU)/mL) present in the inoculum was determined by densitometry and colony counts after serial dilution plating on TSA agar plates. The growth of *E. coli* in liquid (broth) and solid (agar) media was evaluated aerobically at 37 °C. A fresh inoculum was prepared before each experiment to ensure the repeatability of the experimental conditions.

### 2.3. In Vitro Model of the Innate Immune System

THP-1 human monocytes (ATCC^®^ TIB-202TM) were maintained in THP-1 complete medium (THP-1 CCM) and cultured in a controlled atmosphere at 37 °C. Macrophage differentiation was induced by incubation with phorbol myristate acetate (PMA) for 24 h. The culture medium was then replaced, and the cells were left with fresh medium for further 24 h before being exposed to the three functional ingredients, their mixture, and the inflammatory stimulus *E. coli*.

### 2.4. Determination of the Cytotoxicity of the Three Functional Ingredients and Their Mixture

The cytotoxicity of the three functional ingredients and their mixture was evaluated on a model of the THP-1 immune system differentiated into macrophages. Assuming an oral administration route of the mixture, a digestion volume of 2 L was considered to mimic the average volume of liquid contained in the digestive process. Cytotoxicity was measured by constructing a dose-response curve with increasing concentrations of the single functional ingredients and their mixture to identify the maximum usable concentration for subsequent tests. The following concentrations were then tested on THP-1: 50 to 600 μg/mL for PEA, 6.25 to 75 μg/mL for hempseed oil, 1.25 to 15 μg/mL for maritime pine bark dry extract, and 57.5 to 690 μg/mL for the mixture. Based on the results of the dose-response curves, analysis of cell vitality was not performed at higher concentrations due to cell mortality caused by the mixture at the highest concentration tested. The concentrations used for the subsequent experiments were chosen from this concentration range. The three functional ingredients and their mixture were dissolved in THP-1 CCM in absence of antibiotics and in presence of 1.18 mg/mL of bile as surfactant. The suspensions were incubated overnight to increase the solution passage of the functional ingredients and, the following day, the THP-1 cells were washed with Hank’s Balanced Salt Solution (HBSS) and pre-treated for 2 h with the different concentrations of the individual functional ingredients and their mixture. At the end of this incubation, the pro-inflammatory stimulus *E. coli* (1 × 10^6^ CFU/mL) was added to the treated cells for a further 6 h. Cells treated with *E. coli* alone were used as a negative control, while cells treated with 10 μM staurosporine were used as a positive cell death control. At the end of the 8 h treatment, cell vitality was evaluated by ATP assay (PerkinElmer, Waltham, MA, USA) to avoid any possible interference from the bacteria present, which could occur using other tests such as MTS.

### 2.5. Preparation of Conditioned Medium on THP-1 for the Treatment of the Bladder Urothelium Model In Vitro

Based on the results of the cytotoxicity experiments, the THP-1-conditioned medium used to study possible anti-inflammatory activity on the bladder urothelium was obtained by exposing the differentiated THP-1 cells to the concentrations of the three functional ingredients and their mixture for 2 h. The concentrations were 600, 400, 200, 100, 50, and 25 μg/mL for micronized PEA; 75, 50, 25, 12.5, 6.25, and 3.125 μg/mL for hempseed oil; 15, 10, 5, 2.5, 1.25, and 0.625 μg/mL for maritime pine bark dry extract; and 690, 460, 230, 115, 57.5, and 28.75 μg/mL for the mixture. At the end of this incubation, the cells were treated for a further 6 h with the inflammatory stimulus *E. coli* in the presence of the different treatments. Cells exposed only to the inflammatory stimulus in the absence of pre-treatment were used as a positive control of inflammation.

### 2.6. Evaluation of the Anti-Inflammatory Activity of the Three Functional Ingredients and Their Mixture in an In Vitro Bladder Urothelium Model

**Figure 1:**
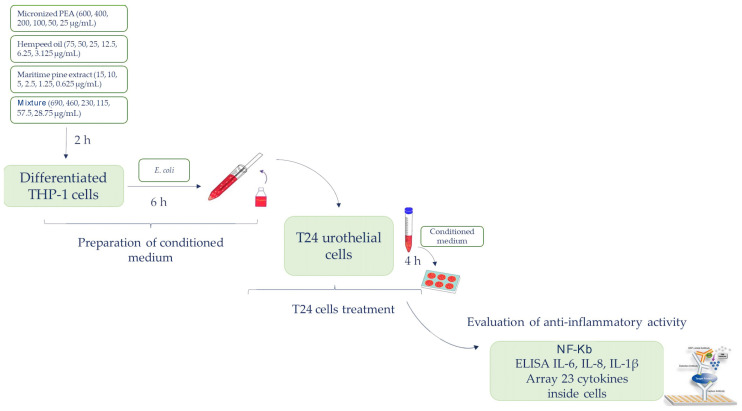
The flow chart illustrating the experimental approach.

The specific anti-inflammatory activity on the bladder urothelium of the three functional ingredients and of the mixture was evaluated on an in vitro bladder urothelium composed of the T24 cell line (ATCC HTB-4TM). T24 cells were exposed for 4 h to conditioned media obtained by treating macrophage-differentiated THP-1 cells with different concentrations of the functional ingredients and mixture in the presence of *E. coli*. At the end of the treatment, T24 cells were detached with trypsin, centrifuged, and lysed by sonication in lysis buffer. The supernatant obtained following centrifugation for 10.000 runs for 10 min at 4 °C was stored at −80 °C. An extensive analysis of the impact of the functional ingredients and mixture on inflammation was conducted by assay for the determination of transcription factor NF-kB (Abcam) and the evaluation of pro-inflammatory cytokine expression with an array of 23 human cytokines (Raybiotech, Peachtree Corners, GA, USA), following the manufacturer’s instructions. The cytokines interleukin 1β (IL-1β), interleukin 6 (IL-6), and interleukin 8 (IL-8) were quantified by ELISA assay. The flow chart shown in Figure 1 illustrates the experimental approach previously described.

### 2.7. Statistical Analyses

The results are reported as fold change calculated as the ratio between values obtained from the respective treatments on inflammation control. As such, the fold change is dimensionless. Experiments were performed in triplicate and results are presented as mean ± standard deviation (SD). Statistical analysis was performed comparing the mixture and each single ingredient for every condition tested, using the *t*-test. OriginLab software (OriginLab Corporation, Northampton, MA, USA) was used for all analyses. A *p*-value of ≤0.05 was considered significant.

## 3. Results

### 3.1. Determination of the Impact of the Three Functional Ingredients and Their Mixture on the Vitality of the In Vitro Model of the Immune System

The PEA content in micronized PEA powder, total lipids in hempseed oil, and procyanidins in maritime pine bark dry extract were determined by HPLC-DAD, spectrophotometric analysis, and gas-chromatography-FID, respectively, and compared with the titer declared in the technical data sheets of the three raw materials (Supplementary Table S2).

**Figure 2:**
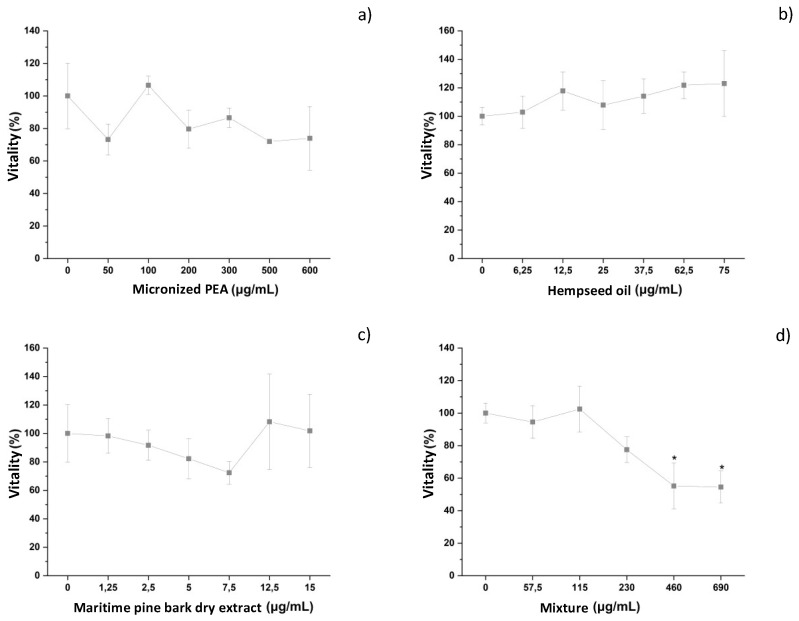
Vitality of differentiated THP-1 cells after treatment with different concentrations of (**a**) micronized PEA, (**b**) hempseed oil, (**c**) maritime pine bark dry extract, and (**d**) mixture. * vitality < 70%.

The cytotoxic activity of the three functional ingredients and of their mixture in the presence of the inflammatory stimulus *E. coli* was evaluated in an innate immune system model in vitro, following the protocol previously described. The three functional ingredients did not induce negative effects on macrophage vitality, as shown in Figure 2a–c, and Table 1. The mixture had a dose-dependent effect on the cell vitality of differentiated THP-1, reaching a *plateau* with a vitality value of less than 70% (54.5 ± 9.9%) relative to the negative control (*E. coli*-treated cells) at a concentration of 690 μg/mL (1380 mg) (Figure 2d and Table 1). Based on the vitality results, the maximum concentration of the mixture used for the experiments was 690 μg/mL, corresponding to micronized PEA at 600 μg/mL, hempseed oil at 75 μg/mL, and maritime pine bark extract at 15 μg/mL (Condition 1). Concentrations higher than this were not investigated due to the mixture-induced effects on vitality, while lower concentrations of the mixture and its functional ingredients were investigated for their anti-inflammatory capacity.

### 3.2. Impact of Conditioned Media on Inflammation of the Bladder Urothelium In Vitro

The specific anti-inflammatory activity on the bladder urothelium of the three functional ingredients and of their mixture was evaluated by exposing the T24 cells to the conditioned media for 4 h. The anti-inflammatory response was investigated by evaluating the effect of the treatments on the nuclear levels of the transcription factor NF-kB, analysis of the release levels of 23 cytokines, and ELISA assays for the cytokines IL-1β, IL-6, and IL-8, which are the ones most involved in inflammatory processes.

**Figure 3:**
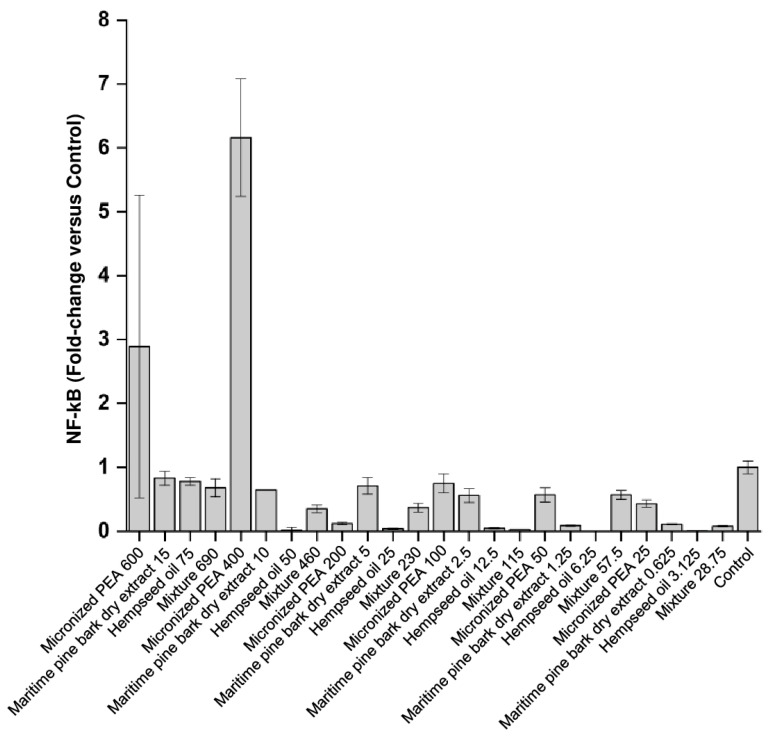
Nuclear levels of the transcription factor NF-kB expressed as fold changes compared to control (cells treated with medium conditioned only with the inflammatory stimulus, *E. coli*) after treatment of bladder urothelium cells with the three functional ingredients and their mixture at different concentrations.

As shown in Figure 3 and Table 2, NF-kB levels were reduced following treatment with hempseed oil, maritime pine bark dry extract, and the mixture, demonstrating their efficacy in reducing the translocation of this pro-inflammatory factor within the cell nucleus, an event that is observed following an inflammatory process. Treatment with micronized PEA from 25 up to 200 μg/mL reduced the nuclear translocation of the transcription factor NF-kB. The mixture was found to be more effective in reducing the nuclear concentration of NF-kB than single functional ingredients at a concentration of 115 μg/mL (Condition 4), and at the maximum concentration used (690 μg/mL, Condition 1).

Supplementary Table S3 shows the expression levels of three cytokines that had a significant change in the treated compared to the control: chemokine growth-regulated (GRO) alpha, GRO a/b/g, and granulocyte-macrophage (GM)-CSF. These cytokines are involved in the inflammatory response, in particular, in the recruitment and activation of the cells of the immune system, ensuring their arrival at the site of inflammation. From these data, it can be seen that the combination of the three functional ingredients studied was effective in reducing the inflammation triggered by the presence of the *E. coli* pathogen. The synergistic action of micronized PEA, hempseed oil, and maritime pine bark dry extract was particularly evident when the mixture was used at concentrations of 57.5 μg/mL (Condition 5) with a value of 0.96 ± 0.00 for GRO a/b/g; 115 μg/mL (Condition 4) with a value of 0.78 ± 0.01 for GRO alpha; 230 μg/mL (Condition 3) with values of 0.65 ± 0.02, 0.51 ± 0.05, and 0.57 ± 0.00 for GRO alpha, GRO a/b/g, and GM-CSF, respectively; 460 μg/mL (Condition 2) with values of 0.58 ± 0.06, 0.58 ± 0.04, and 0.58 ± 0.00 for GRO alpha, GRO a/b/g, and GM-CSF, respectively; and 690 μg/mL (Condition 1) with a value of 0.99 ± 0.03 for GRO alpha.

**Figure 4:**
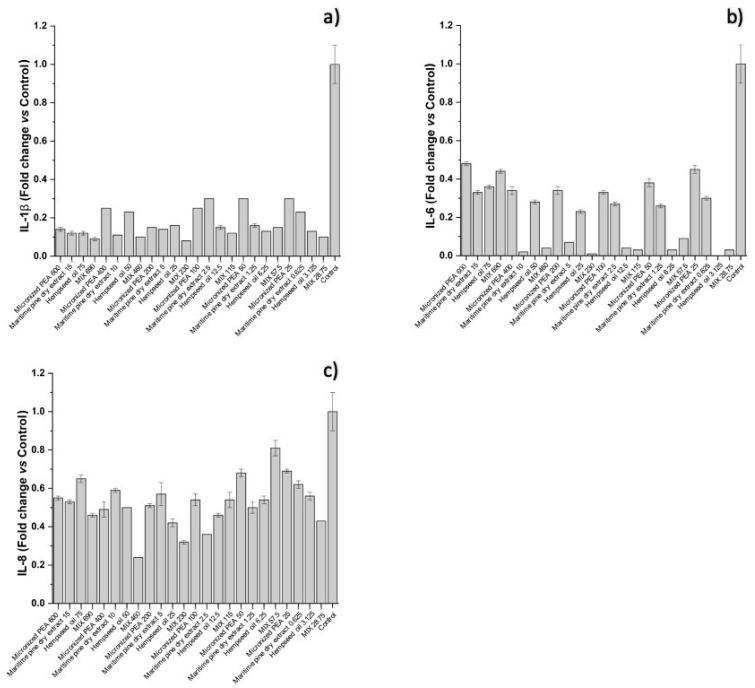
Expression of (**a**) IL-1β, (**b**) IL-6, and (**c**) IL-8, reported as fold changes compared to control (cells treated with medium conditioned only with the inflammatory stimulus, *E. coli*) after treatment of bladder urothelium cells with the three functional ingredients and their mixture at different concentrations.

**Figure 5:**
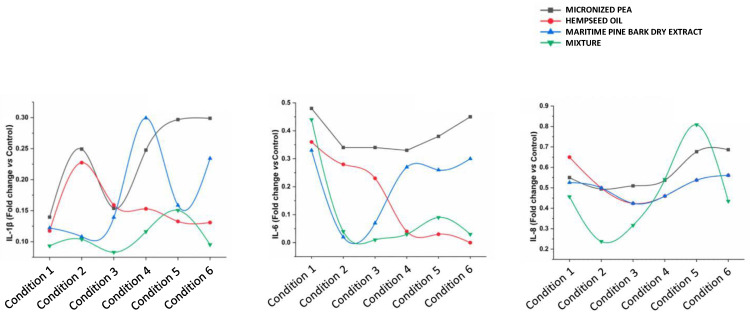
Effects of individual components and their mixture on IL-1β, IL-6, and IL-8 levels in different conditions.

The functional ingredients used individually at the corresponding concentrations did not have the same ability to attenuate the inflammatory process. The cytokines IL-1β, IL-6, and IL-8, which are most involved in inflammatory processes, were studied in more depth by an ELISA assay; the results are reported below. As shown in Figure 4 and Figure 5, and Table 3, treatment with the individual functional ingredients and their mixture caused a reduction in the expression of all cytokines studied. In particular, the mixture resulted in the lowest expression levels of all three cytokines compared to the single functional ingredients at the concentration of 230 μg/mL (Condition 3), with values of 0.08 ± 0.00, 0.01 ± 0.00, and 0.32 ± 0.01 for IL-1β, IL-6, and IL-8, respectively, suggesting a synergistic action of micronized PEA, hempseed oil, and maritime pine bark dry extract. At 115 μg/mL (Condition 4), the mixture was most effective in reducing IL-6, with a value of 0.03 ± 0.00, and IL-1β, with a value of 0.12 ± 0.00. The synergistic action at the highest concentrations of the mixture tested resulted in the reduction of IL-8 expression, with a value of 0.24 ± 0.00 at the concentration of 460 μg/mL (Condition 2), and 0.46 ± 0.01 at the concentration of 690 μg/mL (Condition 1); and in the reduction of IL-1β expression, with a value of 0.10 ± 0.00 at the concentration of 460 μg/mL (Condition 2), and 0.09 ± 0.01 at the concentration of 690 μg/mL (Condition 1).

## 4. Discussion

The treatment of CPP may involve the use of analgesic drugs, anti-inflammatories, and neuro-modulators, which can potentially lead to adverse side effects [1,6]. A strategy that aims to limit drug consumption is represented by formulations based on natural plant extracts, such as hempseed oil and maritime pine bark dry extract, which are well-known for their anti-inflammatory activity. Despite the high variability in its composition according to genotype and environmental factors, hempseed, the edible fruit of the *Cannabis sativa* L. plant and its derivates, is well known for its nutraceutical, cosmetic, and pharmaceutical properties [11,12,16]. It is approximately 25–35% lipids, with a perfectly balanced composition of omega-3 and omega-6 polyunsaturated fatty acids, 20–25% proteins, particularly rich in essential amino acids, and 20–30% carbohydrates, tocopherols, and phytosterols, as well as a large amount of macro- and micro-elements and different bioactive compounds such as phenolics and bioactive peptides [16]. Several in vitro studies have demonstrated neuroprotective, antihypertensive, and hypocholesterolemic activities related to the hempseed’s components, as well as the anti-inflammatory, antimicrobial, and antibacterial effects [16,17,18]. In human studies, the benefit of hempseed oil dietary supplementation has been tested in patients with atopic dermatitis [19], hyperlipidemia [20], and multiple sclerosis [21], as well as in healthy patients to evaluate improvements in serum lipids and glucose profile, oxidation markers, and platelet aggregation [22]. Similarly, maritime pine bark dry extract, which is made by the extraction of the outer bark of *Pinus pinaster* Aiton, is rich in procyanidins, potent anti-inflammatory and antioxidant molecules, suggesting a possible application as a treatment of acute and chronic inflammatory conditions [10,23]. The most extensively studied use of maritime pine bark dry extract is for cardiovascular health, with a demonstrated improvement of endothelial function, chronic venous insufficiency, hypertension and its complications, and coronary artery disease [23,24,25,26]. Finally, PEA is an endogenous lipid produced by microglia and mast cells, that modulates their activation, thereby playing a role in neuroinflammation events [13,27]. Peroxisome proliferator-activated receptor alpha (PPAR-α) represent the molecular target that mediates the main neuroprotective, anti-inflammatory, and analgesic effects of PEA, along with endocannabinoid-mediated mechanisms (entourage effect); these features distinguish PEA from classical steroidal and non-steroidal anti-inflammatory drugs that act by inhibiting the cascade of arachidonic acid [27]. To improve tissue exposure and the dissolution rate following oral administration, micronized and ultra-micronized PEA formulations are currently used, and achieved significant benefits [28]. Specifically, their effectiveness has been demonstrated in the treatment of pain syndromes such as sciatica, peripheral neuropathies, diabetic neuropathies, and post-herpetic neuralgia, as well as pelvic pain, osteoarthritis, headaches, and post-operative pain [13,15,29].

In the bladder, the response to the inflammation caused by micro-organisms such as *E. coli* is mainly mediated by resident macrophages, which released several cytokines that are responsible for the inflammation of urothelial cells [30,31]. The in vitro model used in this study is based on two human cell lines representative of the innate immune system (THP-1) and the urothelium (T24s). The monocytic/macrophage cell line THP-1 represents one of the most used in vitro models for the study of the anti-inflammatory activity of formulations [32]. Similarly, the T24s line is the cell line most used for the study of efficacy and safety of formulations and drugs at the urothelial level [33].

In the present study, we demonstrated that the mixture composed of micronized PEA, hempseed oil, and maritime pine bark dry extract had a synergistic anti-inflammatory effect at different concentrations, with greater effectiveness than that of its individual components. Specifically, the mixture reduces (i) the nuclear content of the transcription factor NF-kB at the concentrations of 115 μg/mL and 690 μg/mL; (ii) the expression of the pro-inflammatory cytokine GRO-alpha in the concentration range 115 to 690 μg/mL; (iii) the expression of the pro-inflammatory cytokine GRO a/b/g at concentrations of 57.5 μg/mL, 230 μg/mL, and 460 μg/mL; (iv) the expression level of the cytokine GM-CSF at the concentrations of 230 μg/mL and 460 μg/mL; (v) IL-1β expression in the concentration range 115 to 690 μg/mL; (vi) IL-6 expression in the concentration range 115 to 230 μg/mL; and (vii) IL-8 expression in the concentration range 230 to 690 μg/mL.

The synergistic anti-inflammatory efficacy of the mixture was particularly evident for the cytokines IL-8 and IL-1β. The cells of the bladder urothelium express but do not normally secrete IL-8. The release of IL-8 occurs in response to inflammatory events affecting the urinary tract, caused, for example, by pathogenic micro-organisms, as in the case of *E. coli*. The greater reduction in the levels of this cytokine following treatment with the studied mixture compared to the individual functional ingredients supports the presence of a synergistic action of the formulation at the level of the bladder urothelium.

The data obtained show that micronized PEA has a dose-independent anti-inflammatory effect on IL-6 expression, while maritime pine bark dry extract and hempseed oil have an inversely dose-dependent effect. The former contributes to anti-inflammatory action at the highest concentrations tested (Conditions 2 and 3, which correspond to the concentrations of 10 and 5 μg/mL); the latter has a greater effect, when used at the lowest concentrations (Conditions 4, 5, and 6, which correspond to 12.5, 6.25, and 3.125 μg/mL). The synergistic reduction of the IL-6 of maritime pine bark dry extract and hempseed oil within the mixture is greater in Condition 3 (fold change 0.01), which corresponds to a concentration equal to 230 μg/mL. Similar effects were also observed for IL-1β. The three functional ingredients taken individually reduce IL-8 in a dose-independent manner, while, when combined, they create a synergistic effect with an increased anti-inflammatory activity for IL-8 under Conditions 1, 2, and 3, corresponding to the mixture concentrations of 690, 460, and 230 μg/mL.

Taken together, the mixture is characterized by an anti-inflammatory activity at the level of the urothelium, derived from the synergy of its functional ingredients. Although in vitro models can provide useful information about the mechanism of action of active ingredients and formulations, the following limitations should be acknowledged. First, considering that the mixture will be administered orally, it will undergo exposure to the digestive process. In this context, we cannot predict the stability of each ingredient, not the effect on the formulation of different biochemical environments encountered during the digestive process. For example, the mixture will undergo exposure to the digestive process and colonic fermentation by the microbial community and intestinal microflora. Among the various tasks performed by this diverse community of micro-organisms is the fermentation of compounds not processed during digestion, such as fibers. In addition, the literature reports the role of fermentation in the inactivation or activation of numerous active ingredients including procyanidins which, post-fermentation, appear to increase in the bloodstream, with a consequent possible increase in their anti-inflammatory action. Therefore, the action of maritime pine bark dry extract and its contribution to the mixture could be amplified by the gut microbiota during the digestive process. Additionally, we do not know what fraction of the ingredients, individual or combined in the formulation, will be available for absorption at the intestinal level (bio-accessibility and bio-availability), or if there will be an alteration of the active ingredients due the hepatic first pass. Second, although our in vitro model reproduces the acute inflammation process, it is not designed to simulate conditions of chronic inflammation. Currently, chronic inflammation cannot be reproduced in vitro. The latter is a long-lasting process simultaneously involving many cell types and biological processes, tissue destruction, and tissue repair attempts. Third, CPP is also related to the inflammation of the nerves present in the pelvic area; in the in vitro model, the neuronal component is not present. Consequentially, while it is possible to draw conclusions about the anti-inflammatory effectiveness of the ingredients and their mixture in the urothelium, it is not possible to evaluate the same activity in nervous tissue.

## 5. Conclusions

The mixture of micronized PEA, hempseed oil, and maritime pine bark dry extract (Pelvipea^®^) at 230 μg/mL showed the greatest efficacy in urothelial IL-1β, IL-6, and IL-8 reduction compared with the individual ingredients. The mixture was also more effective in reducing the NF-kB nuclear concentration compared to the individual functional ingredients at concentrations of 115 μg/mL and 690 μg/mL. This formulation could represent a promising therapeutic option to relieve painful symptoms originating in the bladder. Therefore, in vivo studies are needed to confirm the anti-inflammatory effect of Pelvipea^®^ on the urothelial mucosa in the context of pelvic pain management.

## 6. Patents

The present investigation allows the registration of the patent-pending invention “Pelvipea” (ITALY-PATENT OF INVENTION Question N. 102022000021057 of 12 October 2022-Nutraceutical or pharmaceutical composition for the treatment of pelvic pain-Erbozeta S.p.a.), which deals with the treatment of pelvic pain. Chiara Pastorelli and Roberto Zavaglia are co-inventors of the patent-pending invention “Pelvipea”.

## Figures and Tables

**Table 1:** Vitality of differentiated THP-1 cells after treatment with different concentration of micronized PEA, hempseed oil, maritime pine bark dry extract, and their mixture. * vitality < 70%.

Micronized PEA (μg/mL)	Vitality (%)
0 (control)	100 ± 20.2
50	73.2 ± 9.5
100	106.5 ± 5.6
200	79.6 ± 11.6
300	86.5 ± 5.9
500	71.9 ± 1.3
600	73.9 ± 19.5
**Hempseed Oil (μg/mL)**	**Vitality (%)**
0 (control)	100 ± 6.1
6.25	102.8 ± 11.1
12.5	117.7 ± 13.3
25	107.8 ± 17.1
37.5	114.1 ± 12.1
62.5	121.8 ± 9.4
75	122.9 ± 23.2
**Maritime Pine Bark Dry Extract (μg/mL)**	**Vitality (%)**
0 (control)	100 ± 20.2
1.25	98.3 ± 12.1
2.5	91.7 ± 10.6
5	82.2 ± 14.0
7.5	72.4 ± 7.9
12.5	108.2 ± 33.5
15	101.8 ± 25.7
**Mixture (μg/mL)**	**Vitality (%)**
0 (control)	100 ± 6.1
57.5	94.5 ± 10.0
115	102.5 ± 14.0
230	77.5 ± 7.9
460	55.2 ± 14.1 *
690	54.5 ± 9.9 *

**Table 2:** Nuclear levels of the transcription factor NF-kB expressed as fold changes compared to control (cells treated with medium conditioned only with the inflammatory stimulus, *E. coli*) after treatment of bladder urothelium cells with the three functional ingredients and their mixture at different concentrations. * indicates statistical significance set at *p* < 0.05 for the comparison between the mixture and each ingredient singularly taken.

	Treatment (μg/mL)	NF-kB (Fold Change vs. Control)
Condition 1	Micronized PEA 600	2.89 ± 2.37
	Hempseed oil 75	0.78 ± 0.06
	Maritime pine bark dry extract 15	0.83 ± 0.11
	Mixture 690	0.68 ± 0.14
Condition 2	Micronized PEA 400	6.16 * ± 0.92
	Hempseed oil 50	0.02 ± 0.04
	Maritime pine bark dry extract 10	0.65 * ± 0.00
	Mixture 460	0.35 ± 0.06
Condition 3	Micronized PEA 200	0.12 ± 0.02
	Hempseed oil 25	0.04 ± 0.01
	Maritime pine bark dry extract 5	0.71 * ± 0.13
	Mixture 230	0.37 ± 0.07
Condition 4	Micronized PEA 100	0.75 * ± 0.15
	Hempseed oil 12.5	0.05 * ± 0.01
	Maritime pine bark dry extract 2.5	0.56 * ± 0.11
	Mixture 115	0.03 * ± 0.00
Condition 5	Micronized PEA 50	0.57 ± 0.11
	Hempseed oil 6.25	0.00 ± 0.00
	Maritime pine bark dry extract 1.25	0.09 ± 0.01
	Mixture 57.5	0.57 ± 0.07
Condition 6	Micronized PEA 25	0.43* ± 0.06
	Hempseed oil 3.125	0.01 ± 0.00
	Maritime pine bark dry extract 0.625	0.11* ± 0.01
	Mixture 28.75	0.08 ± 0.01

**Table 3:** Expression of IL-1β, IL-6, and IL-8, reported as fold changes compared to control (cells treated with medium conditioned only with the inflammatory stimulus, *E. coli*) after treatment of bladder urothelium cells with the three functional ingredients and their mixture at different concentrations. * indicates statistical significance set at *p* < 0.05 for the comparison between mix and each ingredient singularly taken.

	Treatment (μg/mL)	IL-1β (Fold Change vs. Control)	IL-6 (Fold Change vs. Control)	IL-8 (Fold Change vs. Control)
Condition 1	Micronized PEA 600	0.14 * ± 0.01	0.48 * ± 0.01	0.55 * ± 0.01
	Hempseed oil 75	0.12 * ± 0.01	0.36 ± 0.01	0.65 * ± 0.02
	Maritime pine bark dry extract 15	0.12 * ± 0.01	0.33 ± 0.01	0.53 * ± 0.01
	Mixture 690	0.09 ± 0.01	0.44 ± 0.01	0.46 ± 0.01
Condition 2	Micronized PEA 400	0.25 * ± 0.00	0.34 * ± 0.01	0.49 * ± 0.04
	Hempseed oil 50	0.23 * ± 0.00	0.28 * ± 0.01	0.50 * ± 0.00
	Maritime pine bark dry extract 10	0.11 ± 0.00	0.02 ± 0.00	0.59 * ± 0.01
	Mixture 460	0.10 ± 0.00	0.04 ± 0.00	0.24 ± 0.00
Condition 3	Micronized PEA 200	0.15 * ± 0.00	0.34 * ± 0.02	0.51 * ± 0.01
	Hempseed oil 25	0.16 * ± 0.00	0.23 * ± 0.01	0.42 * ± 0.02
	Maritime pine bark dry extract 5	0.14 * ± 0.00	0.27 * ± 0.01	0.57 * ± 0.06
	Mixture 230	0.08 ± 0.00	0.01 ± 0.00	0.32 ± 0.01
Condition 4	Micronized PEA 100	0.25 * ± 0.00	0.33 * ± 0.01	0.54 ± 0.03
	Hempseed oil 12.5	0.15 * ± 0.01	0.04 ± 0.00	0.46 ± 0.01
	Maritime pine bark dry extract 2.5	0.30 * ± 0.00	0.27 * ± 0.01	0.36 ± 0.00
	Mixture 115	0.12 ± 0.00	0.03 ± 0.00	0.54 ± 0.04
Condition 5	Micronized PEA 50	0.30 * ± 0.00	0.38 * ± 0.02	0.68 ± 0.02
	Hempseed oil 6.25	0.13 ± 0.00	0.03 ± 0.00	0.54 ± 0.02
	Maritime pine bark dry extract 1.25	0.16 ± 0.01	0.26 * ± 0.01	0.50 ± 0.03
	Mixture 57.5	0.15 ± 0.00	0.09 ± 0.00	0.81 ± 0.04
Condition 6	Micronized PEA 25	0.30 * ± 0.00	0.45 * ± 0.02	0.69 * ± 0.01
	Hempseed oil 3.125	0.13 * ± 0.00	0.00 ± 0.00	0.56 * ± 0.02
	Maritime pine bark dry extract 0.625	0.23 * ± 0.00	0.30 * ± 0.01	0.62 * ± 0.02
	Mixture 28.75	0.10 ± 0.00	0.03 ± 0.00	0.43 ± 0.00

## Data Availability

Data were provided upon request by the Corresponding Author.

## Supplementary Materials

The following supporting information can be downloaded at: https://www.mdpi.com/article/10.3390/cells12040616/s1, Table S1: Description of the samples analyzed. Table S2: Comparison between the titer measured and the titer declared for PEA, procyanidins, and total lipids in the three raw materials. Table S3: Expression of GRO alfa, GRO a/b/g, and GN-CSF reported as fold-changes compared to control (cells treated with medium conditioned only with the inflammatory stimulus, *E. coli*) after treatment of bladder urothelium cells with the three functional ingredients and their mixture at different concentrations. * indicates statistical significance set at *p* < 0.05 for the comparison between the mixture and each ingredient singularly taken.
