# Supplementary material for: Micronized Palmitoylethanolamide, Hempseed Oil, and Maritime Pine Bark Dry Extract (Pelvipea®) for Pelvic Pain: An In Vitro Study for Urothelial Inflammation Treatment
Source: Cells. 2023 Feb 14;12(4):616. doi: 10.3390/cells12040616 (PMC9953806; doi:10.3390/cells12040616)
Supplement: Supplementary file 1 [file cells-12-00616-s001.zip › cells-2191830-supplementary.pdf]

Supplementary Table S1. *Description of the samples analyzed.*

Sample	Reference order	State
Micronized PEA powder (EZ00049678)	22LA04095	Powder
Hempseed oil (EZ00049037)	22LA04097	Liquid
Maritime pine bark dry extract (EZ00050328)	22LA04096	Powder
Mixture (VC139)	22LA04073	Powder

Supplementary Table S2. *Comparison between the title measured and the title declared for PEA, procyanidins, and total lipids in the three raw materials.*

Ingredients	Content measured (%)	Content declared (%)
Micronized PEA (PEA)	91.1 ± 9.8	≥ 99
Hempseed oil (total lipids)	99.9	100
Maritime pine bark dry extract (procyanidins)	73.6 ± 0.8	65-75

***Supplementary Table S3**. Expression of GRO alfa, GRO a/b/g, and GN-CSF reported as fold-changes compared to control (cells treated with medium conditioned only with the inflammatory stimulus, E. coli) after treatment of bladder urothelium cells with the three functional ingredients and their mixture at different concentrations. *indicates statistical significance set at p<0.05 for the comparison between the mixture and each ingredient singularly taken..*

	**Treatment (µg/mL)**	**GRO alfa (Fold-change vs. Control)**	**GRO a/b/g (Fold-change vs. Control)**	**GM-CSF (Fold-change vs. Control)**
**Condition 1**	Micronized PEA 600	1.49* ± 0.06	1.14 ± 0.04	1.01 ± 0.08
	Hempseed oil 75	1.43* ± 0.03	1.56* ± 0.06	1.21 ± 0.02
	Maritime pine bark dry extract 15	1.79* ± 0.03	1.91* ± 0.02	1.39 ± 0.02
	Mixture 690	0.99 ± 0.03	1.19 ± 0.06	1.10 ± 0.21
**Condition 2**	Micronized PEA 400	0.79* ± 0.04	0.92* ± 0.08	1.10 *± 0.11
	Hempseed oil 50	1.04* ± 0.03	0.98* ± 0.05	1.56* ± 0.09
	Maritime pine bark dry extract 10	0.71 ± 0.16	0.97* ± 0.13	0.81* ± 0.14
	Mixture 460	0.58 ± 0.06	0.58 ± 0.04	0.58 ± 0.00
**Condition 3**	Micronized PEA 200	1.28* ± 0.05	0.81 ± 0.23	0.90 ± 0.26
	Hempseed oil 25	0.90* ± 0.13	0.88* ± 0.04	1.36* ± 0.01
	Maritime pine bark dry extract 5	1.00* ± 0.09	1.22* ± 0.07	1.19* ± 0.04
	Mixture 230	0.65 ± 0.02	0.51 ± 0.05	0.57 ± 0.00
**Condition 4**	Micronized PEA 100	1.23* ± 0.13	0.58 ± 0.37	0.21 ± 0.26
	Hempseed oil 12.5	1.17* ± 0.09	1.17* ± 0.08	1.71* ± 0.02
	Maritime pine bark dry extract 2.5	1.69* ± 0.28	1.71* ± 0.27	1.82* ± 0.16
	Mixture 115	0.78 ± 0.01	0.72 ± 0.02	0.79 ± 0.01
**Condition 5**	Micronized PEA 50	0.91 ± 0.03	1.00 ± 0.08	1.15 ± 0.11
	Hempseed oil 6.25	1.23* ± 0.03	1.16* ± 0.07	1.47* ± 0.05
	Maritime pine bark dry extract 1.25	1.75* ± 0.16	1.44* ± 0.22	1.29 ± 0.14
	Mixture 57.5	0.97 ± 0.09	0.96 ± 0.00	1.31 ± 0.05
**Condition 6**	Micronized PEA 25	1.17 ± 0.01	1.13 ± 0.00	1.19 ± 0.3
	Hempseed oil 3.125	1.19 ± 0.04	1.20 ± 0.07	1.52 ± 0.05
	Maritime pine bark dry extract 0.625	0.66 ± 0.03	0.77 ± 0.07	1.55 ± 0.07
	Mixture 28.75	1.33 ± 0.15	1.35 ± 0.10	1.39 ± 0.10
